# Bibliometric analysis of global research on breast cancer with HER2-low expression

**DOI:** 10.1007/s12672-025-03820-5

**Published:** 2025-11-18

**Authors:** Junpeng Zhu, Jin Hu, Hao Lei, Runze Li, Yu Zhao, Yaqi Zhao, Hengyu Chen, Lei Li, Chong Lu, Chunping Liu

**Affiliations:** 1 Department of Breast and Thyroid Surgery, Union Hospital, Tongji Medical College, Huazhong University of Science and Technology, Wuhan, 430022 China; 2 Department of Breast and Thyroid Surgery, Renmin Hospital of Wuhan University, Wuhan, 430060 China; 3 Department of Breast and Thyroid Surgery, The Second Affiliated Hospital of Hainan Medical University, Haikou, 570311 China

**Keywords:** Breast cancer, HER2-low expression, Bibliometrics, Molecular pathological characteristics, Clinical prognosis, Research status

## Abstract

**Background:**

The abnormal expression of Human Epidermal Growth Factor Receptor 2 (HER2) is closely related to the prognosis of breast cancer. Since the concept of HER2-low expression was introduced in 2018, researches on HER2-low expression breast cancer have burgeoned. This bibliometric analysis seeks to evaluate the worldwide distribution of literature, research focuses, and upcoming trends in HER2-low expression breast cancer research.

**Methods:**

We extensively searched for articles on HER2-low expression breast cancer from January 2018 to March 2025 using the Web of Science database. By employing the Bibliometrix, VOSviewer and CiteSpace, we systematically examined information regarding countries, institutions, authors, journals, and keywords.

**Results:**

A total of 573 literature on HER2-low expression breast cancer was included. These literature come from 54 countries, 1145 institutions, and 4125 authors. China and the United States emerge as leading contributors to this field, with the Harvard University being the most prolific institution.Curigliano is the most influential scholar in this research domain.The leading journals in terms of literature sources are *CANCERS* and *FRONTIERS IN ONCOLOGY*.The most cited reference is “Trastuzumab Deruxtecan in Previously Treated HER2-Low Advanced Breast Cancer”.Keyword analysis indicates that the research emphasis has shifted towards “prognostic factor”, “immunohistochemistry”, “targeted therapy”, “diagnosis”, “guideline”, “overall survival” and “pathological complete response” in recent years.

**Conclusion:**

This study applies bibliometric tools to analyze the literature on HER2-low expression breast cancer, which shows a notable upward trend and highlights the clinical significance, providing researchers with the priorities and hotspots in this crucial field.

**Supplementary Information:**

The online version contains supplementary material available at 10.1007/s12672-025-03820-5.

## Introduction

Breast cancer is one of the most prevalent malignant tumors among women globally, and its incidence continues to rise annually [1]. Its subtypes are classified based on the expression status of hormone receptors (HR) and human epidermal growth factor receptor 2 (HER2), which are primarily categorized into three major groups: Luminal type (HR-positive, HER2-negative or HER2-positive), HER2 overexpression type (HR-negative, HER2-positive), and triple-negative breast cancer (HR-negative, HER2-negative). HER2 is a member of the epidermal growth factor receptor (EGFR) family. This family is pivotal in cellular signal transduction under normal physiological conditions, regulating processes such as cell proliferation, differentiation, and apoptosis, significantly influencing the biological behavior and pathogenesis of breast cancer.

The increasing importance of HER2-low expression in breast cancer is due to several factors. Firstly, it represents a significant proportion of breast cancer cases, around 45% to 55% [2], HER2 protein expression exists on a spectrum (IHC 0 to 3+), with HER2-low demonstrating intermediate molecular characteristics between HER2-zero and HER-2 positive tumors, which is a large patient population. Secondly, with the development of new targeted therapies, such as antibody-drug conjugates, HER2-low expression has become a potential therapeutic target, offering new treatment options and hope for these patients. Traditionally, breast cancer HER2 expression status has been categorized as either HER2-positive or HER2-negative. However, in recent years, the concept of HER2-low expression has been introduced, initially observed in the NSABP B-47 trial [3], although its specific definition was not yet established. Subsequently, the definition of HER2-low expression was further clarified in the DB-04 trial [4]. According to the 2018 ASCO-CAP guidelines for HER2 testing in breast cancer, HER2-low expression breast cancer is characterized by a HER2 IHC score of 1 + or 2 + along with a negative ISH test result [5]. Current research indicates that HER2-low expression breast cancer accounts for approximately 45–55% of all breast cancer cases [2]. Clinical trials, such as the NSABP B-31 trial and N9831 trial, have suggested potential benefits from adjuvant trastuzumab treatment for these patients with early-stage HER2-low expression breast cancer [6]. Despite the results of the NSABP B-47 trial showed that trastuzumab does not provide any survival benefits for patients with HER2-low expression breast cancer [7], recent developments in antibody-drug conjugates (ADCs) have demonstrated positive outcomes in treating this type of cancer, bringing new possibilities for patients [8]. Landmark antibody-drug conjugate (ADC) trials revealed striking survival differences, DB-04 reveals T-DXd reduced mortality risk by 36% in HER2-low mBC, establishing a new treatment paradigm [4]. In DAISY, ADC efficacy extends to HER2-null tumors via bystander effects, challenging binary biomarker definitions [9]. Furthermore, HER2-low breast cancer exhibits significant biological heterogeneity compared to HER2-zero breast cancer (IHC 0) in terms of molecular subtypes, gene expression profiles, and clinicopathological characteristics [10, 11].

The basic principle of bibliometric methods lies in quantitatively analyzing various elements of literature. It mainly counts and statistically analyzes the external characteristics of documents, such as the number of publications, citations, co-authorship, and keywords. For example, citation analysis assumes that a high citation count indicates the document’s high influence and importance in its field. Co-authorship analysis reveals collaborative relationships among researchers. Keyword analysis helps identify research hotspots and trends. Through these quantitative analyses, bibliometric methods can reflect the development and characteristics of a research field. Despite abundant bibliometric studies in oncology, few have specifically focused on HER2-low breast cancer. Compared to study which offers an in-depth exploration of this field [12], our study adopts a distinct analytical perspective, aiming to deliver actionable insights for future clinicians and researchers. In addition to this, our study’s selection of 2018 as the chronological starting point demonstrates stronger scientific rationale. This decision directly aligns with the landmark 2018 ASCO/CAP guideline update that formally established “HER2-low” as a distinct biological and clinical entity through standardized diagnostic criteria (IHC 1 + or IHC 2+/ISH- status). By anchoring our analysis to this nosologically critical juncture, we capture the paradigm shift in research focus that emerged post-guideline, thereby offering a more contextualized examination of the field’s evolution.

This bibliometrics analysis original articles directly related to HER2-low expression breast cancer published between January 23, 2018, and March 13, 2025. A total of 573 articles were retrieved. Based on this data, a bibliometric analysis was performed to identify the latest research hotspots.

## Materials and methods

### Data source and retrieval strategies

**Fig. 1:**
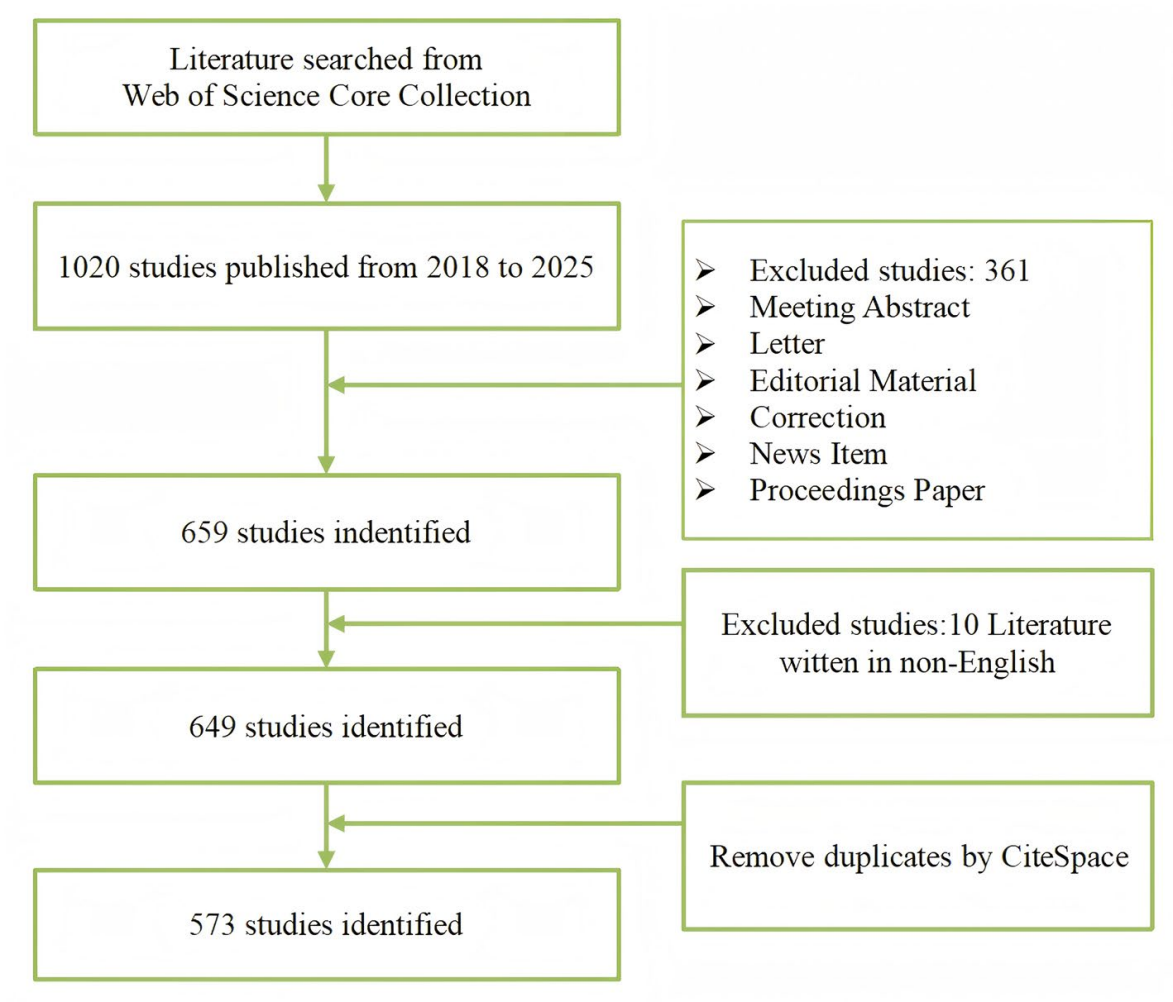
Literature searching strategy

All data were retrieved from the Web of Science Core Collection (WoSCC) database, chosen for its widespread use and acceptance in bibliometric analysis [13]. WoSCC was selected as the sole data source for this bibliometric analysis, based on three primary considerations: (1) WoSCC indexes only high-impact oncology journals, ensuring the dataset represents high-impact, peer-reviewed authoritative publications on HER2-low breast cancer. This aligns with our goal to evaluate the research focuses, and upcoming trends in HER2-low expression breast cancer. (2)As citation analysis is significant to this study, WoSCC provides standardized, fully indexed citation data essential for accurate network modeling—a feature incompletely exists in other databases. (3) WoSCC comprehensively covers landmark clinical trials (e.g., DESTINY-Breast04) and their citation cascades, enabling precise tracking of how pivotal revolution (e.g., ADC therapies) reshaped research focuses. While multi-database approaches expand coverage, they introduce low-correlation literature and inconsistent citation metrics, potentially weaken trends in a rapidly evolving field like HER2-low breast cancer. Our approach follows established precedents in oncology bibliometrics [14, 15]. The database was accessed and data were exported on March 16, 2025 to ensure accuracy and consistency. The search strategy is as follows: (TS=(“breast neoplasm”) OR TS=(“breast cancer”) OR TS=(“breast carcinoma”)) AND (TS=(“HER2-low”) OR TS=(“ERBB2-low”)). To minimize potential biases, the following refinement criteria were applied: the search was restricted to documents published between 2018 and 2025, written in English, and limited to articles and reviews. We exclusively selected original research articles (Articles) and review articles (Reviews) as primary data sources, based on two fundamental rationales: (1) Articles comprise peer-reviewed empirical investigations (e.g., randomized controlled trials, prospective cohorts), offering transparent methodologies and verifiable datasets. (2) Reviews provide systematic syntheses of domain knowledge (e.g., evidence-based meta-analyses), collectively establishing the nosological framework of the discipline. (3) Inclusion of non-archival publication types (e.g., conference abstracts, case reports) would introduce uncontrolled heterogeneity and risk of premature conclusions. In total, 649 documents were retrieved. Remove duplicates by CiteSpace, finally 573 documents were included. Figure 1 illustrates the search strategies and results.

### Data collection and analysis

Download the literature information from the WoSCC database, including titles, authors, publication years, countries/regions, institutions, keywords, citations, abstracts, and references. This information was saved in plain text format with the file named “download.txt”, and imported into Excel. VOSviewer (version 1.6.20) [16], CiteSpace (version 6.2.R6) [17], and the “bibliometrix” package (version 4.2.3, https://www.bibliometrix.org) were utilized for the analysis of information such as countries, authors, institutions, journals, and keywords. We present the results using tables and visualizations.

### Bibliometrics

Bibliometrics utilizes mathematical and statistical techniques to examine the current state, research patterns, and upcoming developments in a particular field using published literature. This analysis includes factors such as the country of origin, institutional affiliations, and authorship [18]. VOSviewer, CiteSpace, and the R package “bibliometrix” are widely used tools for bibliometric analysis. These applications excel at visually representing bibliographic data. VOSviewer constructs network visualizations using nodes and links, where node size corresponds to frequency (e.g., of publications, citations, or keyword occurrences), and node color typically indicates cluster membership. Link thickness and length represent the strength of association between nodes, effectively mapping relationships among countries, authors, institutions, and keywords [19]. CiteSpace offers similar core functionalities to VOSviewer and additionally generates specialized visualizations such as keyword timeline maps, keyword burst detection maps, and dual-map overlays of journal citation networks [20]. Furthermore, the “bibliometrix” R package can produce thematic maps and thematic evolution plots, delineating the evolution of research hotspots over time [21].

## Results

### Analysis of annual publications

**Fig. 2:**
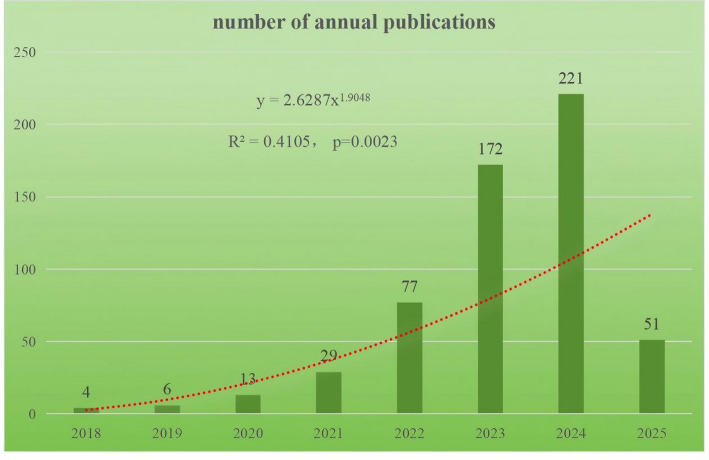
The trend of annual publication number from 2018 to 2025

This study analyzes 573 research papers on HER2-low expression breast cancer published from 2018 onwards. There has been a noticeable rise in publication volume since 2018, with a particularly significant surge seen in 2023. The trend line analysis indicates a correlation, with an R² value of 0.4105. The trend line analysis indicates a correlation, with an R² value of 0.4105 and a P-value of 0.0023, showing a moderate positive relationship between the publication year and the number of publications. This suggests that as time progresses, there is a noticeable increase in the number of publications related to the topic. The moderate R² value indicates that while there is a clear trend, other factors may also influence the number of publications. This could be due to increasing research interest, advancements in the field, or greater awareness of the importance of the topic. Projections indicate that the number of publications in 2025 is estimated to surpass 250. Please refer to Fig. 2 for a detailed visualization.

**Fig. 3:**
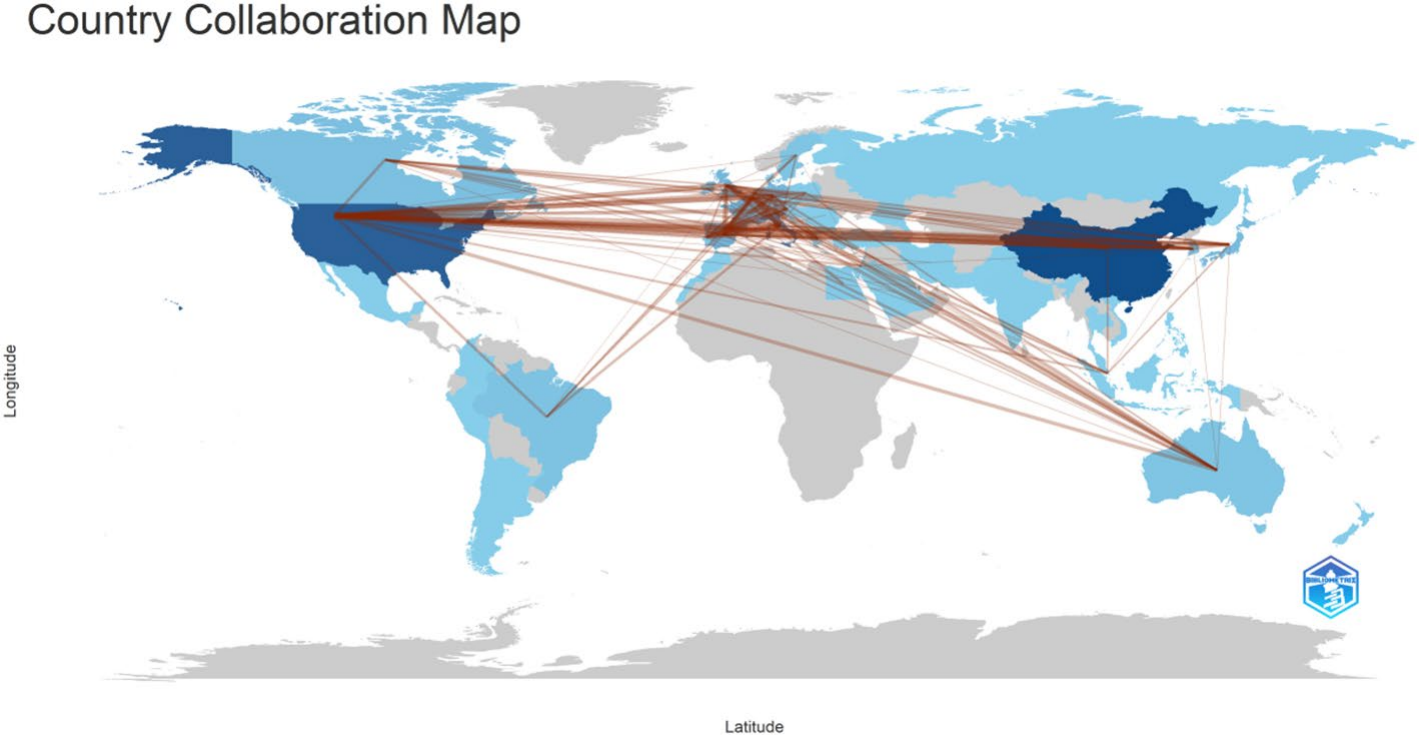
Country cooperation map

### Analysis of countries

Between 2018 and 2025, a total of 54 countries or regions published research on HER2-low expression breast cancer. The top ten countries by publication volume are presented in Table 1, with North America, Asia, and Europe leading the field. China (*n* = 210), the United States (*n* = 184), and Italy (*n* = 100) are the top contributors. The United States has the highest H-index, indicating its significant academic impact in this area. China is the only developing country in the top ten, showing its increasing research output in this field. Figure 3 shows the international collaboration map, highlighting collaborations between countries. The United States has extensive collaborations with multiple nations, with Italy being its closest collaborator. Table 2 shows the frequency of collaboration between countries.

**Table 1:**
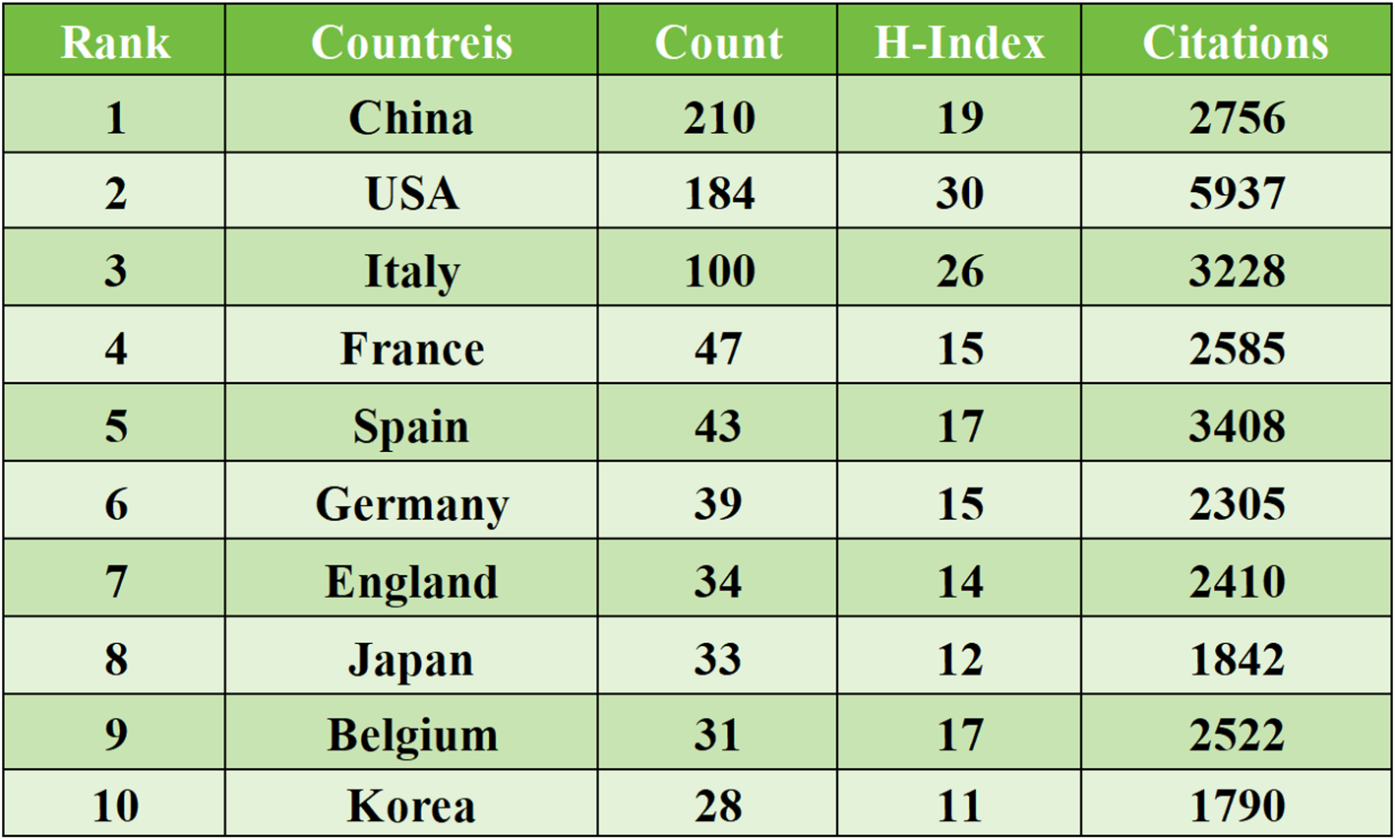
Top 10 countries in terms of publication volume

**Table 2:**
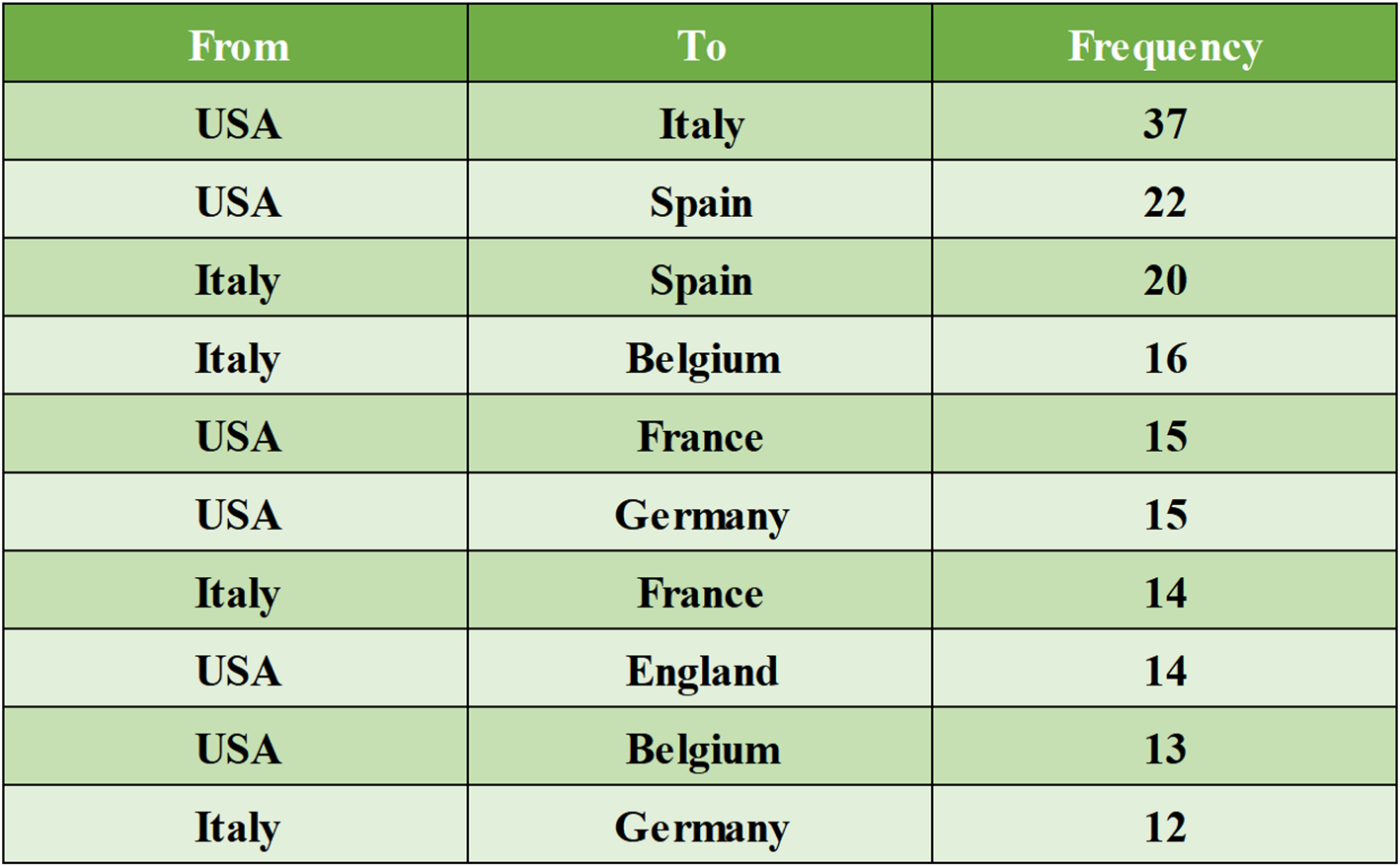
The frequency of collaboration between countries

### Analysis of institutions

**Fig. 4:**
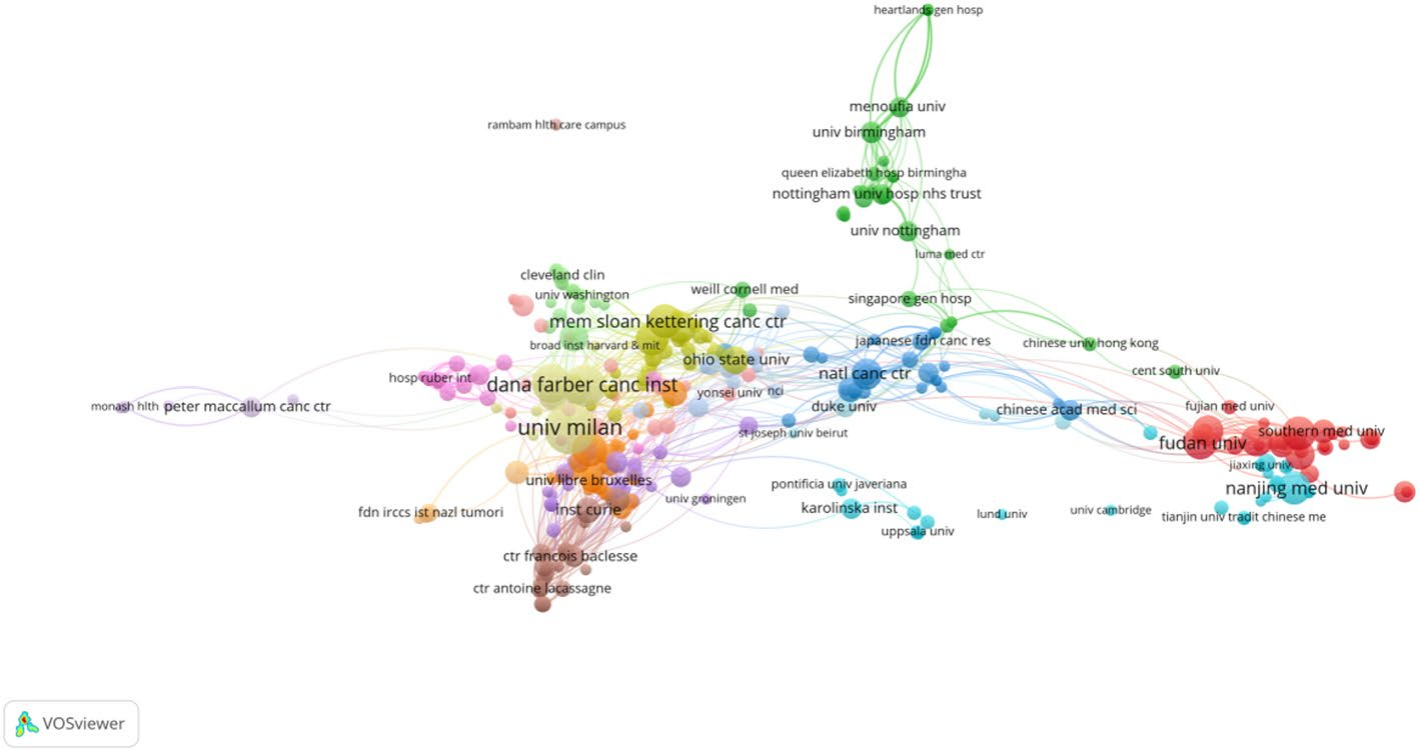
Collaboration map of institutions

A total of 1145 institutions globally have contributed to research on HER2-low expression breast cancer. The top ten institutions, which collectively authored 340 articles (59.33% of all publications), are predominantly located in the United States, China, Italy, and France. As detailed in Table 3, the Harvard University (*n* = 47), University of Milan (*n* = 45), and the UNICANCER (*n* = 40) emerge as the leading institutions in terms of publication volume. Among these top institutions, the IRCCS European Institute of Oncology and University of Milan boast the highest H-index, while the UNICANCER leads in citation counts, underscoring their significant academic impact in this field. The collaboration among Université Libre de Bruxelles, Università degli Studi di Milano, Dana-Farber Cancer Institute, Memorial Sloan Kettering Cancer Center is depicted in Fig. 4, illustrating their strong partnership. Additionally, there is a robust partnership between Southern Medical University, Fudan University, and Nanjing Medical University. This demonstrates the global nature of HER2-low breast cancer research, characterized by: (1) Co-led pivotal trials (e.g., TROPICS-02). (2) Shared core technologies (e.g., spatial transcriptomics via INSERM-Dana-Farber partnerships). (3) Internationally endorsed guidelines (e.g., 2024 ESMO recommendations with voting experts from 10 top institutions). This suggests that more breakthroughs in this field can be expected in the future.

**Table 3:**
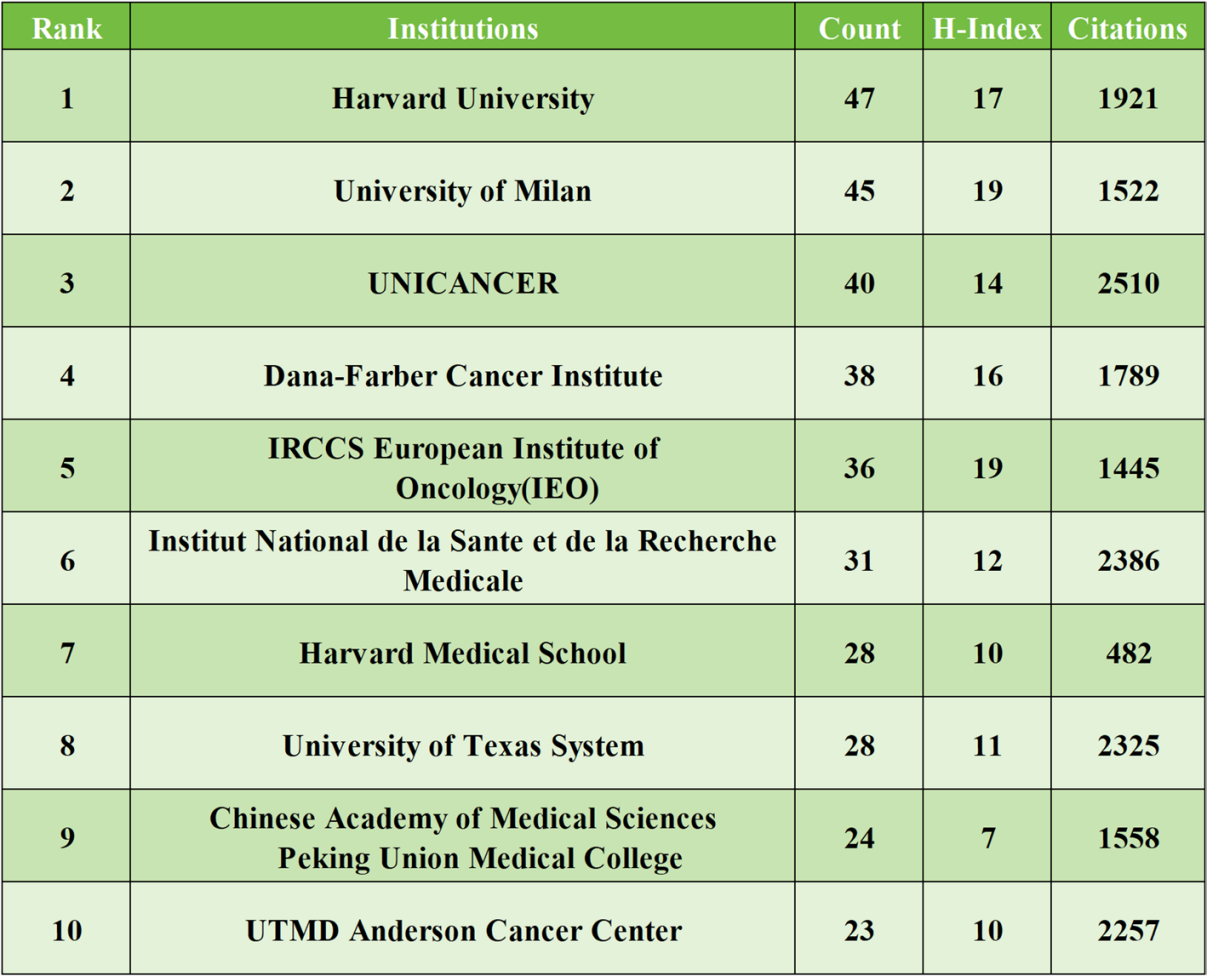
Top 10 institutions in terms of publication volume

### Analysis of authors

**Fig. 5:**
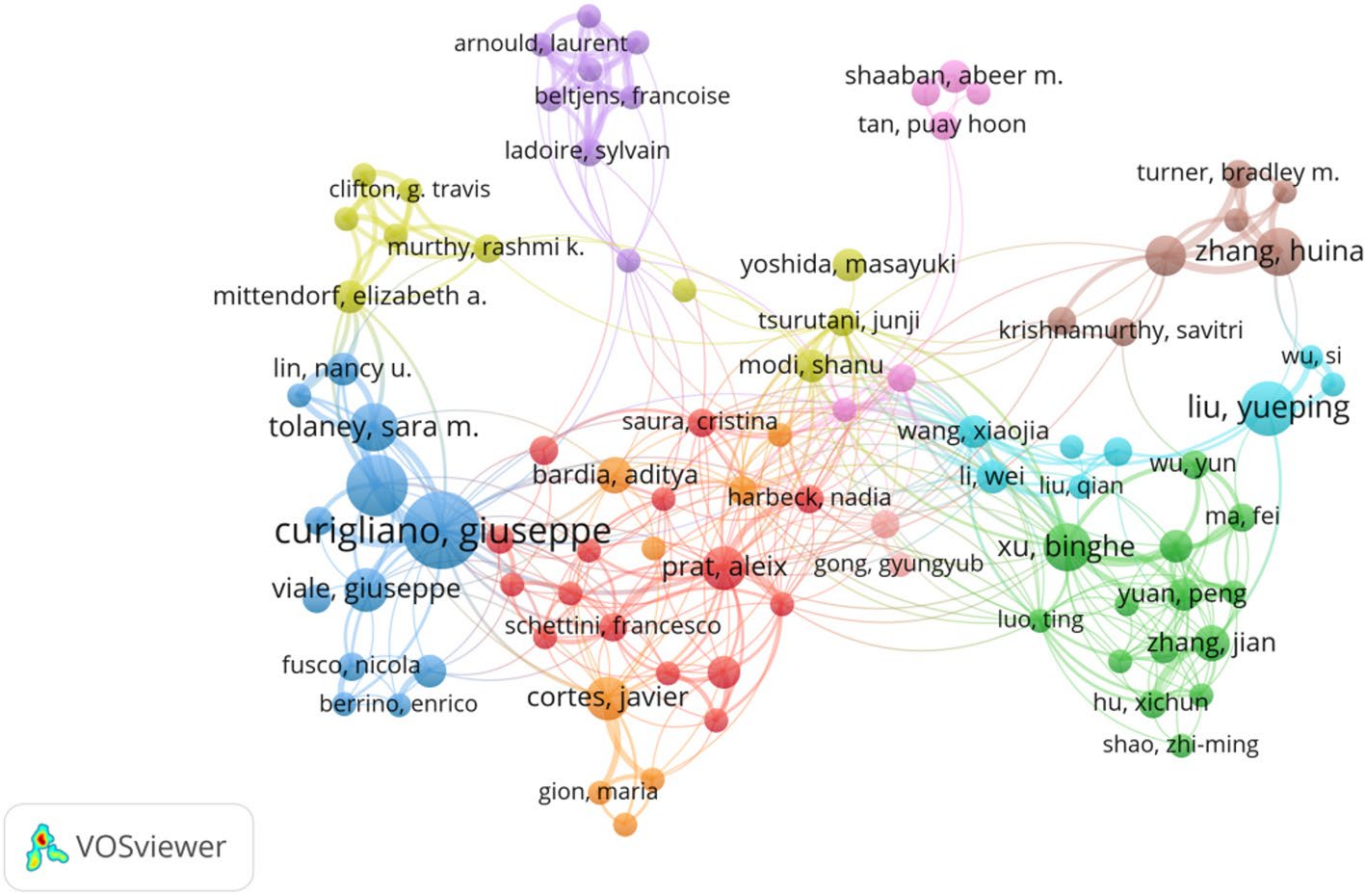
Visualization map of authors

A total of 4125 authors have contributed to research on HER2-low expression breast cancer. The top three authors by publication volume are Curigliano, Giuseppe (*n* = 27), Tarantino, Paolo (*n* = 18), and Tolaney, Sara M (*n* = 13). Detailed in Table 4 are the publication volume, citation count, and H-index of the top ten authors, highlighting Curigliano, Giuseppe as the most prolific contributor in this field. Curigliano, Giuseppe’s recent research focuses on establishing pathological detection standards, exploring ADC drug applications, investigating genomic characteristics, and assessing prognostic implications related to HER2-low expression breast cancer [22–25]. Figure 5, depicting author collaboration patterns, reveals predominantly domestic collaborations with limited international partnerships.

**Table 4:**
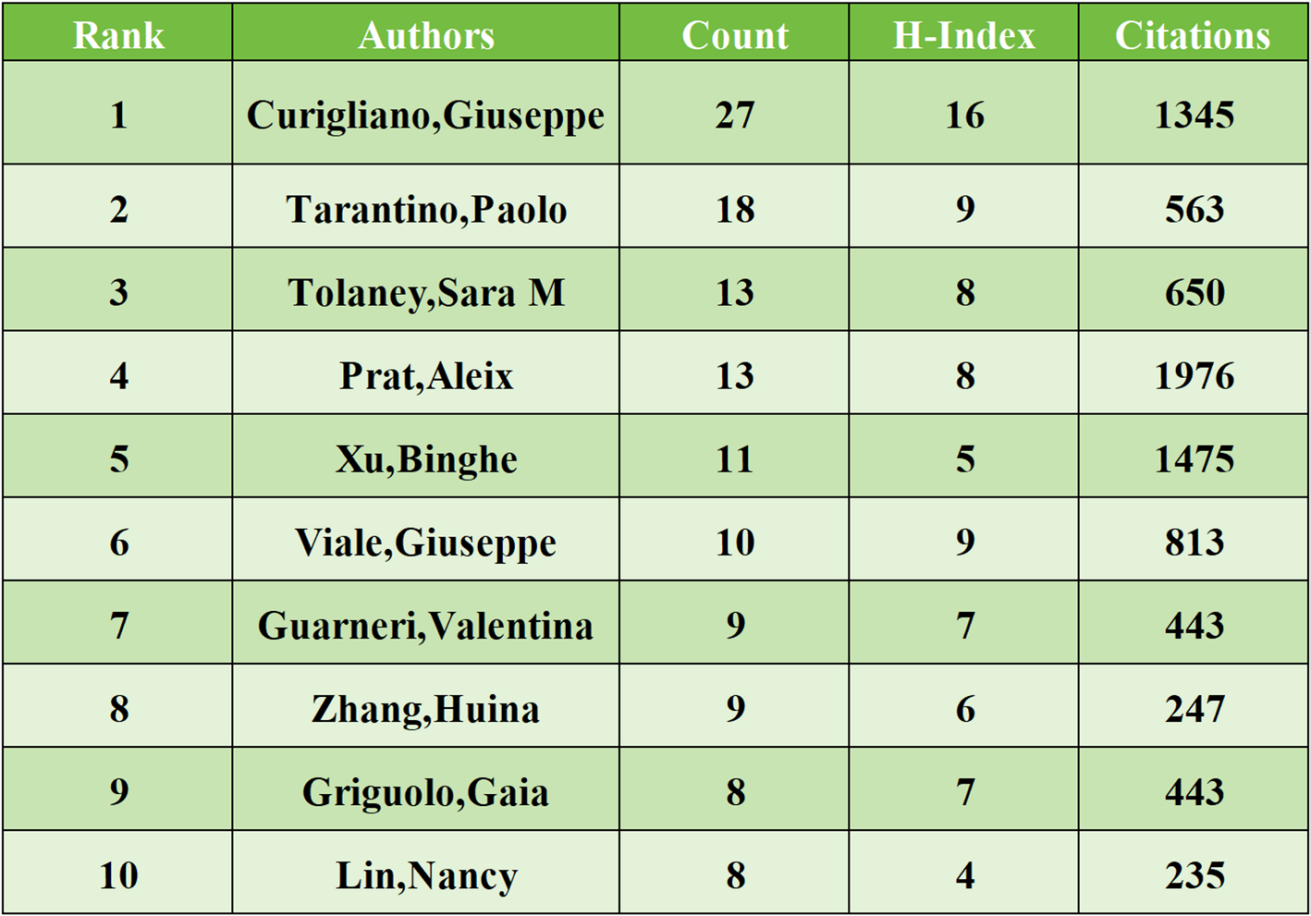
Top 10 authors in terms of publication volume

### Analysis of journals

**Fig. 6:**
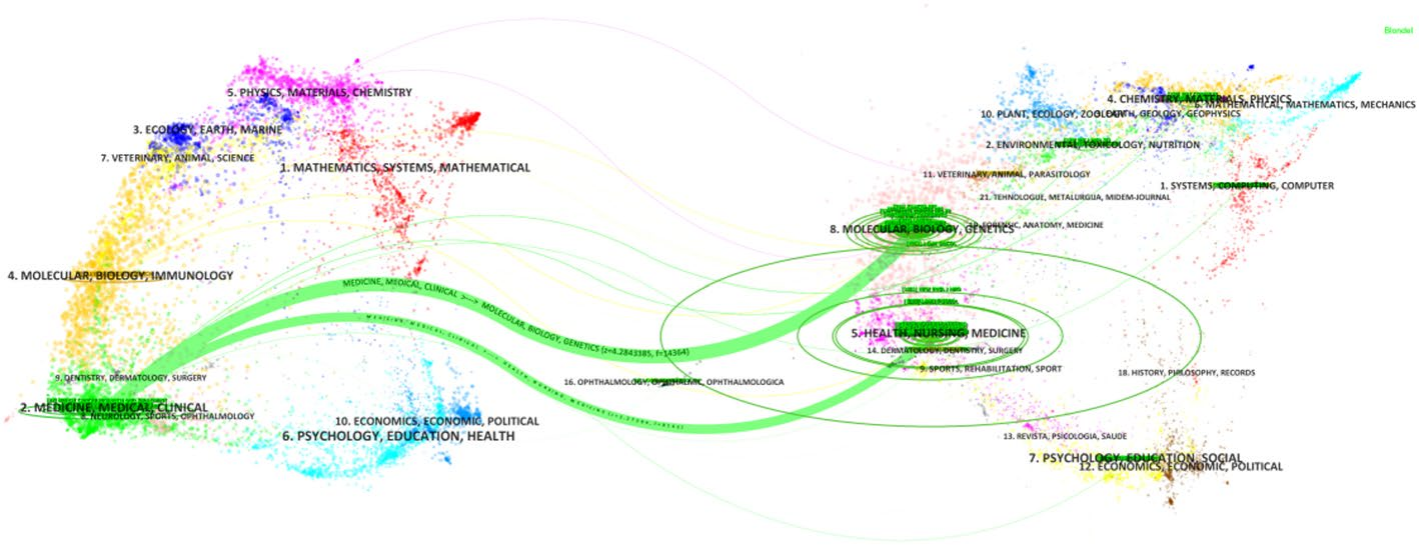
Overlay map of citing journals and cited journals

A total of 209 journals have published articles on HER2-low expression breast cancer. Among the top ten journals by publication volume, four specialize in breast cancer. The journal with the highest impact factor is the *EUROPEAN JOURNAL OF CANCER*, which had an impact factor of 7.6 in 2024. The top three journals by publication volume are *CANCERS* (*n* = 31), *FRONTIERS IN ONCOLOGY* (*n* = 26), and *BREAST CANCER RESEARCH AND TREATMENT* (*n* = 24); details are provided in Table 5. In terms of citation count, the top three journals are *JOURNAL OF CLINICAL ONCOLOGY* (*n* = 2953), *NEW ENGLAND JOURNAL OF MEDICINE* (*n* = 1451), and *ANNALS OF ONCOLOGY* (*n* = 1071); refer to Table 6 for specifics. Both the top ten journals by literature sources and the most cited journals predominantly fall within the Q1 category of the Journal Citation Reports (JCR), underscoring the significant clinical impact of HER2-low expression breast cancer research. To visually illustrate the relationship between citing and cited journals, we employed CiteSpace to generate an overlay graph (Fig. 6). The dual-map overlay of journals is a visualization tool in bibliometrics that integrates two different maps into one. It allows for the comparison of journal citation patterns across different fields or time periods. This overlay helps researchers identify commonalities and differences in citation networks, revealing interdisciplinary connections and shifts in research focus. It provides a comprehensive view of the intellectual structure and dynamics of a research area, aiding in understanding the evolution and integration of knowledge. The graph reveals two primary citation pathways within HER2-low breast cancer research: The dominant path is literature in clinical medicine frequently cites foundational work in molecular biology and genetics. This dominant pathway underscores how clinical advancements are increasingly built upon fundamental biological discoveries, include:1)Clinical trials (e.g., DESTINY-Breast04) cite mechanistic studies on HER2 protein trafficking to explain drug mechanisms like T-DXd’s bystander effect.2)Guideline updates (e.g., ASCO/CAP 2023) incorporate findings from spatial transcriptomics research to refine HER2-low diagnostic thresholds. The Secondary path is Literature in clinical medicine cites research focused on healthcare systems, nursing, and implementation science. This secondary pathway highlights the reliance of clinical application on healthcare delivery research. For example, Toxicity management protocols in clinical studies reference nursing research on ILD monitoring frameworks.

**Table 5:**
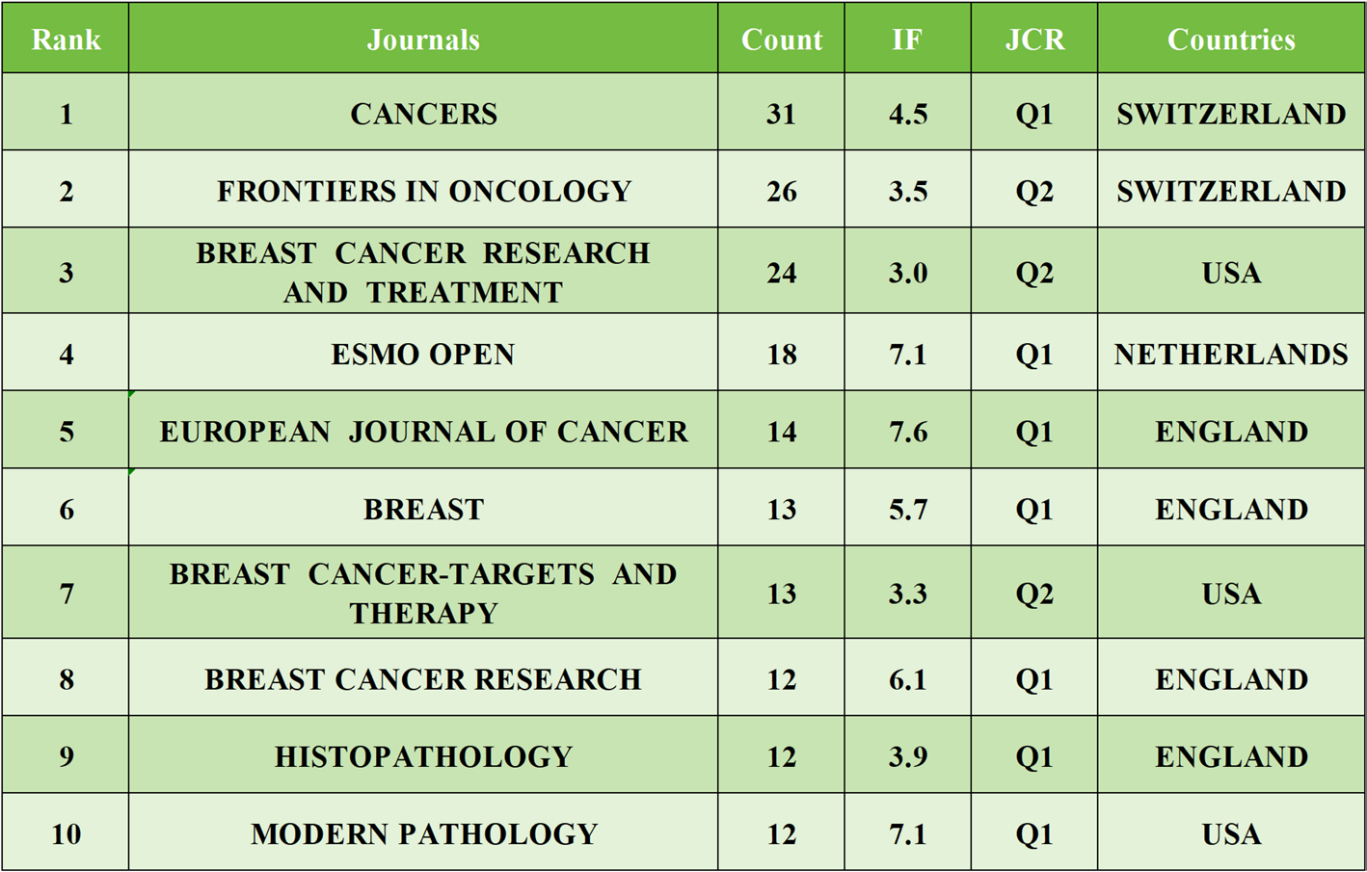
The top 10 journals in terms of literature sources

**Table 6:**
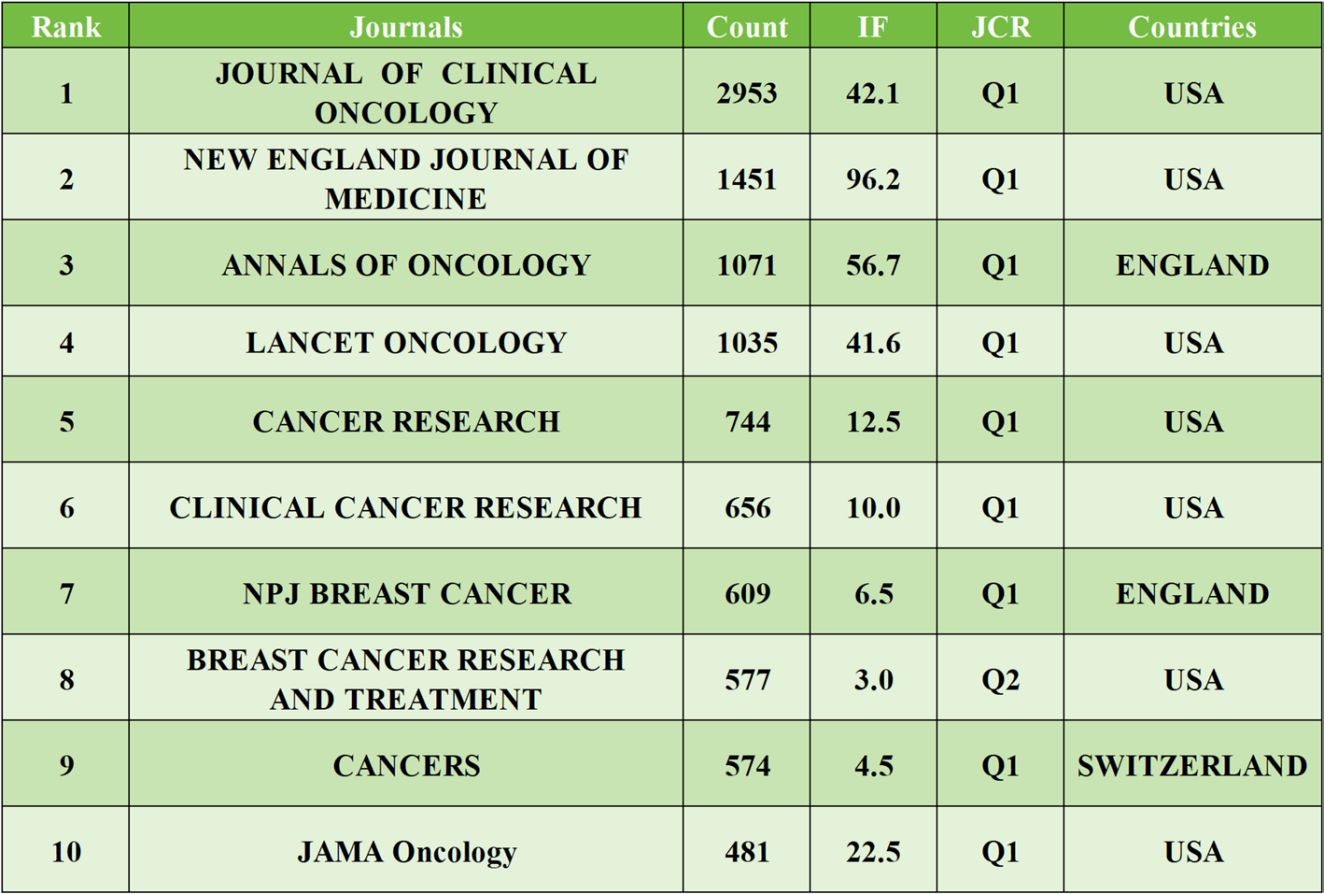
The top 10 Journals in terms of citation count

### Analysis of references

Among all the references, the top three most cited articles are as follows: “Trastuzumab Deruxtecan in Previously Treated HER2-Low Advanced Breast Cancer” (*n* = 1344), “Antitumor Activity and Safety of Trastuzumab Deruxtecan in Patients with HER2-Low-Expressing Advanced Breast Cancer: Results From a Phase Ib Study” (*n* = 546), and “HER2-Low Breast Cancer: Pathological and Clinical Landscape” (*n* = 462), as shown in Table 7. It is notable that the top ten most cited articles are all from 2019 to 2023.

**Table 7:**
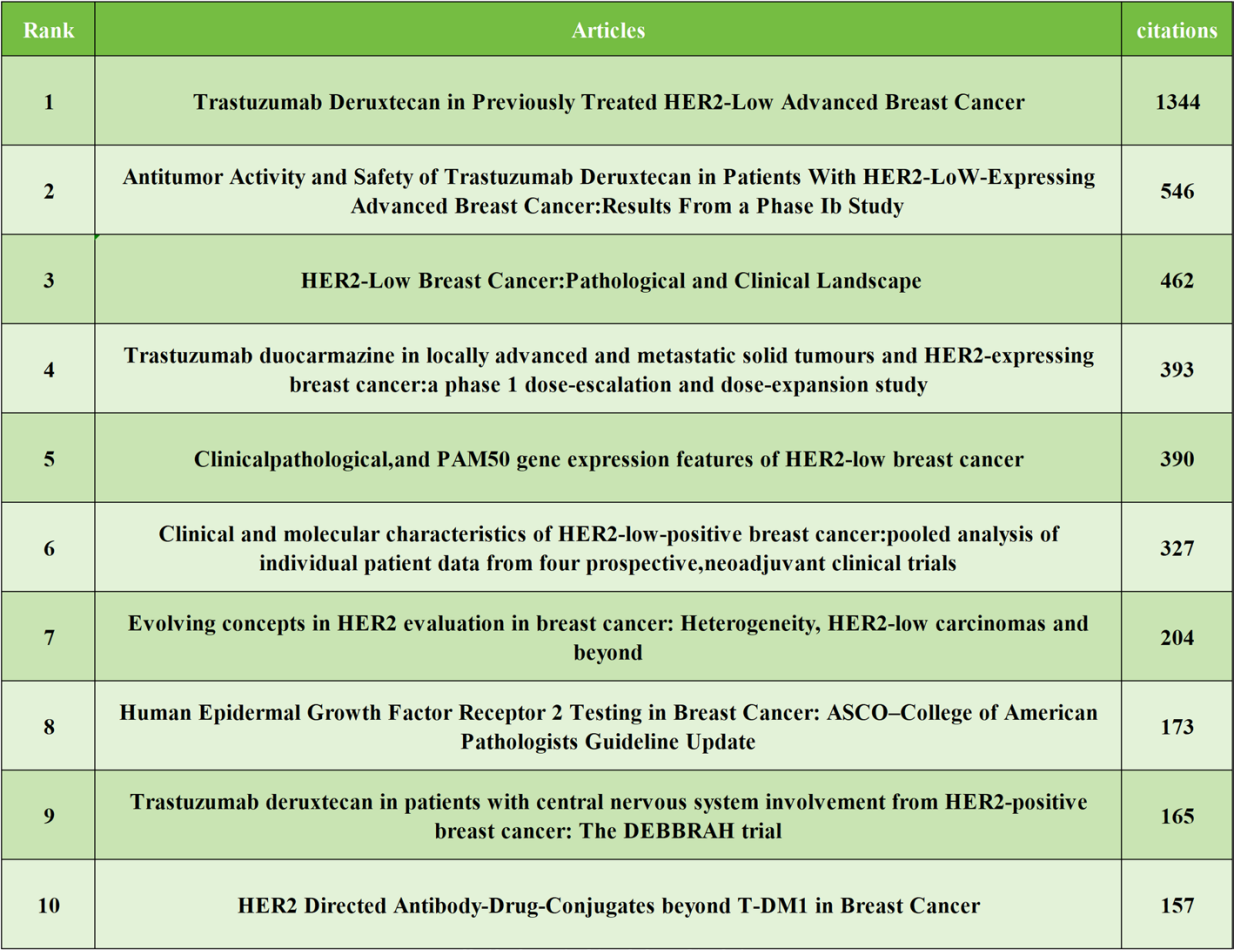
The top 10 most cited literatures

### Analysis of keywords

**Fig. 7:**
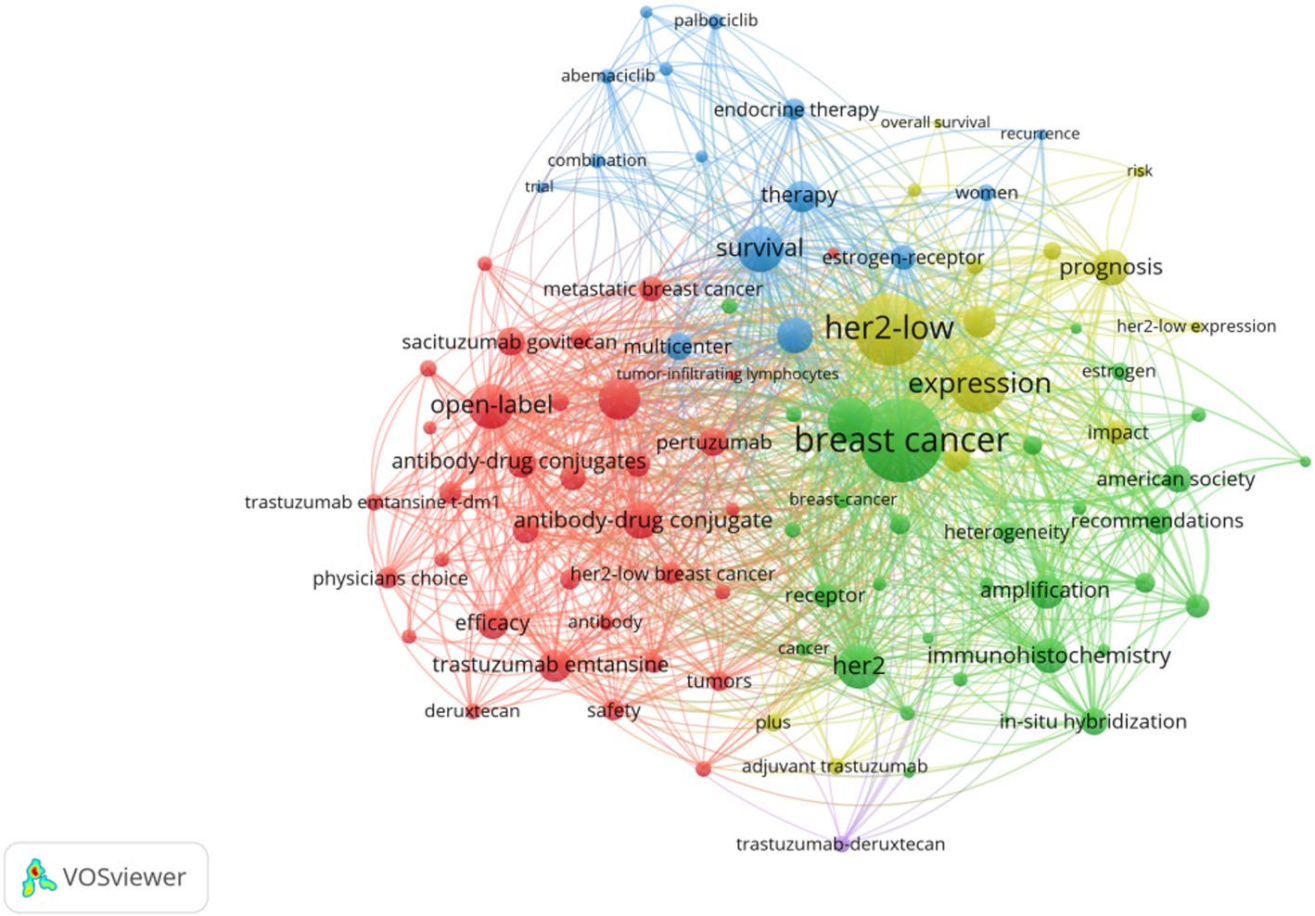
Co-occurrence map of keywords

**Fig. 8:**
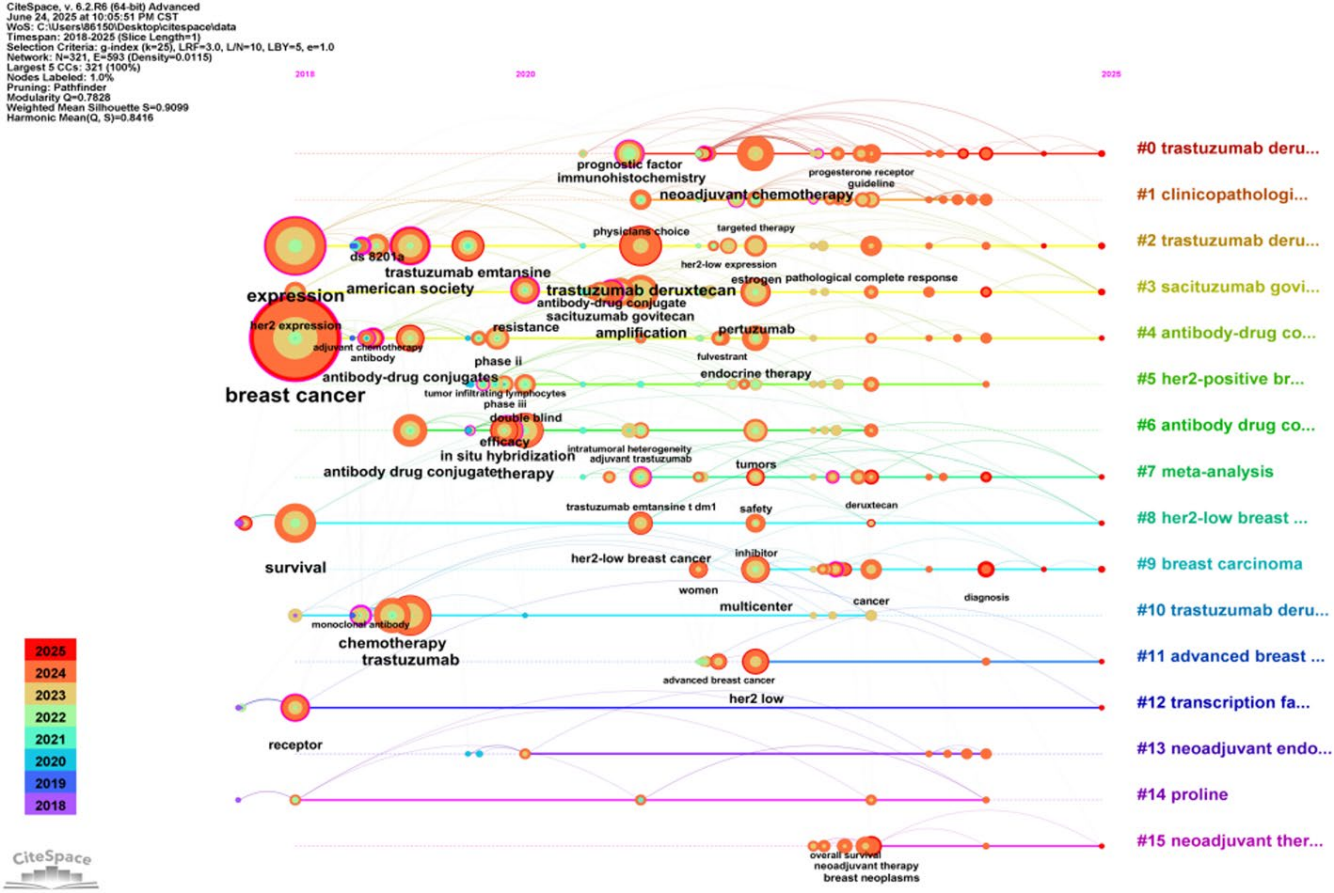
Timeline map of keywords

**Fig. 9:**
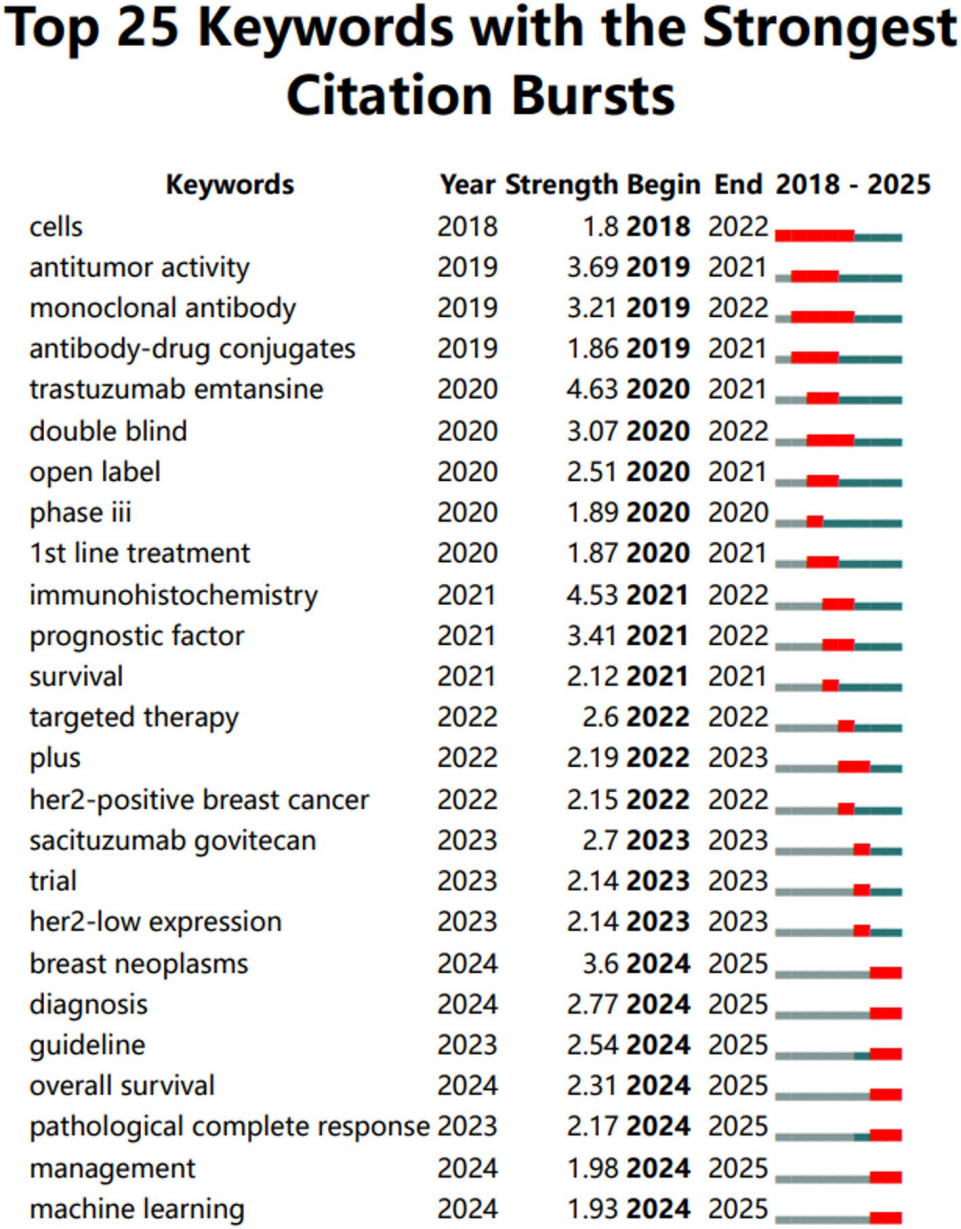
Top 25 Keywords with the strongest citation burst

**Fig. 10:**
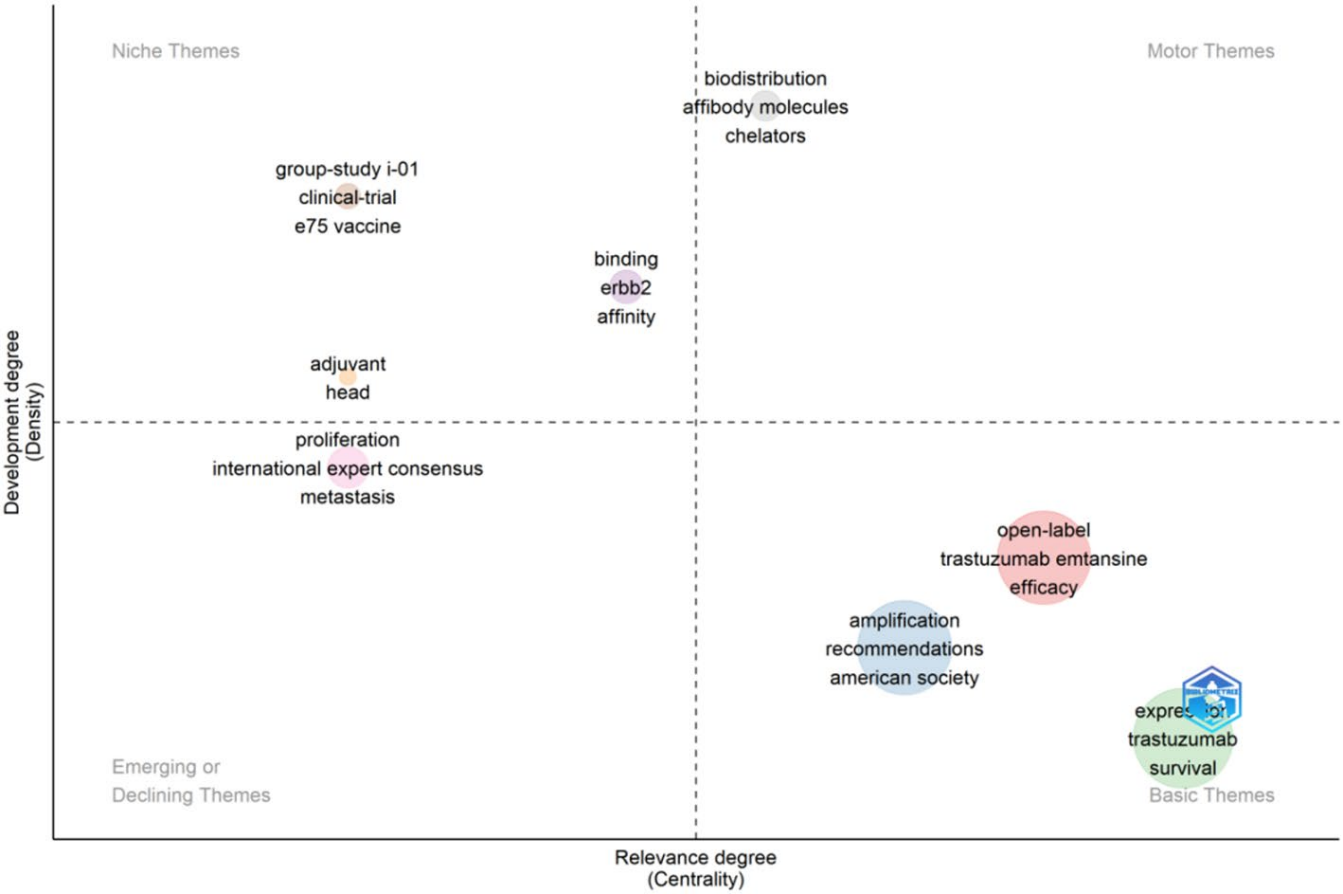
Distribution map of themes

Keyword analysis provides valuable insights into the main themes and emerging topics in a particular field. By analyzing keywords extracted from 573 relevant studies, we can identify distinct research directions in Fig. 7. One key area of focus is treatment modalities, including endocrine therapy, targeted therapy, and chemotherapy. Other directions include ADC drugs, prognosis, and molecular pathological features. To visualize recent trends in keywords, we utilized CiteSpace to generate a keyword-timeline graph (Fig. 8) and a keyword burst graph (Fig. 9). These graphs illustrate a historical shift in research focus: initially centered around “antibody-drug conjugates”, “antitumor activity”, “drug clinical trials” and “first-line treatment”, recent years have seen a transition towards keywords such as “prognostic factor”, “immunohistochemistry”, “overall survival” and “pathological complete response”. The keyword burst graph highlights the top 25 keywords with significant bursts, shown in red during periods of rapid growth. Trastuzumab emtansine stands out for its high burst intensity, highlighting the significance of ADC drugs in HER2-low expression breast cancer. Furthermore, by utilizing the “bibliometrix” package, we constructed a thematic map (Fig. 10), that showcases basic research areas in the low-right quadrant, such as survival traits of HER2-low expression breast cancer and clinical trials involving ADC drugs.

## Discussion

HER2-low expression breast cancer is characterized by a HER2 IHC score of 1 + or 2 + and a negative ISH test result. Various research studies have suggested that individuals with breast cancer showing low levels of HER2 expression have diverse outcomes in terms of survival rates and response to chemotherapy when compared to those with HER2 0 expression [26–29]. There is still a significant amount of disagreement over whether HER2-low expression should be considered as a separate clinical subtype. Although recent studies have not definitively confirmed HER2-low expression as an independent subtype, it remains a valuable tool for guiding treatment decisions [30].

Bibliometric analysis of literature related to HER2-low expression breast cancer in the WoSCC database from 2018 to 2025 reveals a steady increase in publications following the concept’s official introduction in 2018. A total of 573 articles have been identified, involving 54 countries, 1,145 institutions, and 4,125 authors. The United States maintains a dominant research impact due to its high incidence of breast cancer [31]. However, China is making strides in both publication volume and research quality, standing out as the only developing country in the top ten. The top research institutions by publication volume align closely with the leading countries, showcasing strong collaborative relationships. The journal with the highest number of published articles is *CANCERS*, which covers a wide range of topics in cancer research, including biology, treatment, and prevention. This journal is particularly important for advancing research on personalized breast cancer treatment, especially in relation to HER2-low expression breast cancer. One of the most cited studies in this field, “Trastuzumab Deruxtecan in Previously Treated HER2-Low Advanced Breast Cancer,” was published in the prestigious *NEW ENGLAND JOURNAL OF MEDICINE*. This study highlighted the significant benefits of Trastuzumab Deruxtecan in improving progression-free and overall survival in HER2-low expression breast cancer patients, drawing attention to ADC drugs as a promising area of research. While China and the United States lead in terms of publication volume, it is worth noting the significant contributions from Italian researchers, with the top two authors in terms of publication volume hailing from Italy. Professor Giuseppe Curigliano, from the University of Milan and the European Institute of Oncology, has significantly advanced breast cancer drug development. His impactful publication “Antibody-drug conjugates: Smart chemotherapy delivery across tumor histologies” in *CA-A CANCER JOURNAL FOR CLINICIANS* with an impact factor of 503.1 discusses clinical ADC drug applications. The top ten most cited articles focus on clinical research aspects including precise diagnosis [2], molecular pathology [32], prognosis, and treatment [33] of HER2-low expression breast cancer.

Current research hotspots concerning HER2 features of HER2-low expression breast cancer. The previous classification system for HER2 categorized it as either positive (HER2 IHC3 + or IHC2 + and ISH positive) or negative [34]. As a result, both HER2-low expression and HER2-0 expression were classified as HER2 negative. However, recent studies have suggested that HER2-low expression presents distinct clinicopathological features compared to HER2-0 expression breast cancer. Research by Schettini F et al. suggested that HER2-low expression was more likely to have axillary lymph node metastasis than HER2-0 expression breast cancer [32]. Moreover, research has also revealed notable disparities in survival rates and recurrence rates between breast cancer with low HER2 expression and breast cancer with HER2-0 expression. A study conducted by Ergun Y et al. suggested that individuals with early breast cancer, regardless of hormone receptor status, experience longer disease-free survival (DFS) and overall survival (OS) if they had low HER2 expression instead of HER2-0 expression [35]. Yang et al.‘s research suggested that in early breast cancer, patients with HER2-low expression had a more favorable prognosis than those with HER2-0 expression breast cancer, regardless of hormone receptor status [36]. However, some studies indicate that there are no statistically significant differences in recurrence rates and survival rates between HER2-low expression and HER2-0 expression groups. Kang et al.‘s research indicates that hormone receptor-positive patients undergoing neoadjuvant chemotherapy did not exhibit a statistically significant difference in OS and DFS between those with low HER2 expression and those with HER2-0 expression [37]. Furthermore, Viale et al.‘s research proposed that there is no notable difference in clinicopathological features and prognosis between breast cancer patients with low HER2 expression and those with HER2-0 expression [38]. Hence, it is recommended that future researchers delve deeper into studies to elucidate the variances in clinicopathological characteristics and prognosis between the two groups.

Recent research has also been focusing on the genomics and molecular characteristics of HER2-low expression. A previous study suggested that HER2-low expression shows distinct gene expression patterns compared to HER2-0 expression in breast cancer. Hui T et al.‘s research demonstrates that, compared to HER2-0 expression breast cancer, HER2-low expression has a higher positive rate of HR and lower levels of Ki67. Additionally, the HR status may influence the prognosis of patients with HER2-low expression, and patients with HR-positive/HER2-low expression may have a favorable outcome [39].

The recent clinical trial emphasized the notable effectiveness of Antibody-Drug Conjugate (ADC) medications in managing patients with HER2-low expression breast cancer. ADCs are a type of targeted therapy medication made up of monoclonal antibodies with precise antigen binding abilities and powerful cytotoxic drugs [40]. Recent research has also been focusing on the treatment of HER2-low breast cancer [12]. They reviewed 13 clinical trials related to HER2-low breast cancer patients who included information on intervention, recruitment and the main measurement indices. PFS and AEs are the main indices measured in clinical trials, and researchers are currently concerned about the efficacy and safety of ADC drugs in HER2-low patients. We can also see from the graph that the main clinical trials currently revolve around HER2-low advanced/metastatic breast cancer. Several phase 3 clinical trials involving T-DXd and other commonly used chemical drugs have been conducted, and the results suggest the therapeutic potential and advantages of T-DXd in treating HER2-low breast cancer. The FDA’s approval of Mylotarg (gemtuzumab ozogamicin) in 2000 marked the inception of ADC drugs, which were initially focused on hematologic malignancies, such as leukemia and Hodgkin`s lymphoma. In recent years, researchers have expanded ADC development to include solid tumors such as breast cancer, lung cancer, and gastric cancer [41]. Trastuzumab Deruxtecan (T-DXd) is a significant breakthrough as it is the first HER2-targeted ADC to demonstrate substantial clinical effectiveness and safety in breast cancer patients with low HER2 expression [42]. The research findings on HER2-low expression in breast cancer align significantly with recent changes in clinical guidelines, particularly in the context of treatment paradigms. The 2024 CSCO Breast Cancer Guidelines have dedicated a separate section to HER2-low expression, emphasizing the clinical significance of trastuzumab deruxtecan (T-DXd) for treating advanced HER2-low breast cancer. This reflects the growing recognition that patients with HER2-low disease can benefit from targeted therapies, as demonstrated by the DESTINY-Breast04 trial, which showed substantial improvements in progression-free survival and overall survival.

The DESTINY-Breast04 study is a landmark clinical trial that has significantly advanced our understanding of HER2-low breast cancer. This research demonstrated that patients with HER2-low breast cancer can benefit from targeted therapies, such as T-DXd (trastuzumab deruxtecan), which was previously considered effective only for HER2-positive cancers. By highlighting the therapeutic potential in the HER2-low subgroup, DESTINY-Breast04 has expanded the treatment landscape for breast cancer and underscored the importance of redefining HER2 status in clinical practice. Our study builds on these findings by further exploring the biological mechanisms and clinical implications of HER2-low expression in breast cancer, aiming to provide additional insights that could enhance patient stratification and treatment strategies in this emerging therapeutic area. Research priorities shifted from disease characterization to targeted therapy post-DESTINY-Breast04.Prior to the DESTINY-Breast04 (DB-04) trial results, the research landscape was dominated by efforts to standardize HER2-low definition/detection methodologies and elucidate prognostic implications. This reflects a phase dedicated to delineating the biological essence of HER2-low breast cancer and addressing the pressing demand for effective therapies. Following DB-04’s publication, a marked thematic transition occurred. “Antibody-drug conjugates (ADCs)”, “clinical trials” and “treatment response” emerged as dominant burst terms and core research themes. This shift directly reflects DB-04 rapidly redirected scientific inquiry toward efficacy validation, mechanistic investigations, optimization strategies, and overcoming resistance mechanisms of targeted therapies (particularly ADCs).The 2023 joint update of HER2 testing guidelines by the American Society of Clinical Oncology (ASCO) and the College of American Pathologists (CAP) introduced substantive refinements in pathological interpretation and clinical management for HER2-low breast cancer. Concurrently, the European Society for Medical Oncology (ESMO) issued an expert consensus encompassing four critical dimensions: include biological characteristics, pathological diagnosis, clinical treatment and clinical trial design of HER2-low breast cancer. Following the DESTINY-Breast04 (DB-04) trial, both the volume of Phase III ADC trials and associated research funding surged globally. This trajectory unequivocally demonstrates how DB-04 has propelled a worldwide transformation in the HER2-low breast cancer therapeutic landscape.DESTINY-Breast04 marks a significant advancement in ADC drugs for breast cancer by targeting the HER2-low expression range. This breakthrough disrupts the conventional “binary” treatment approach of anti-HER2 therapies and has shown promising outcomes. It offers the first successful anti-HER2 targeted therapy for individuals with HER2-low expression breast cancer [43]. Currently, there is ongoing clinical research to incorporate new ADC drugs for the treatment of HER2-low expression breast cancer, offering a range of potential treatment options. However, the development of ADCs still faces numerous challenges, such as intricate pharmacokinetics, potential side effects, and the issue of drug resistance [44]. As a result, there is an anticipation for the development of ADC drugs that offer improved safety and efficacy in the future.

As new ADC drugs are being slowly introduced for the treatment of breast cancer with HER2-low expression, it is crucial to differentiate between HER2-0 and HER2-low expression in order to determine the most suitable treatment strategy. The differentiation between HER2 0 and HER2 1 + staining intensity in immunohistochemistry can sometimes be subtle, and the subjective interpretations of pathologists may affect the consistency of HER2 assessments. Hence, it is crucial to establish standardized HER2 testing protocols in order to prevent inaccurate treatment choices.

The research trends on HER2-low breast cancer are increasingly influencing future healthcare policies and resource allocation. As studies continue to validate the therapeutic potential of targeted treatments for HER2-low patients, policymakers are likely to prioritize funding for further clinical trials and biomarker research. This will drive the development of more precise diagnostic tools and treatment protocols, ensuring that patients with HER2-low breast cancer receive optimal care. Additionally, resource allocation may shift towards supporting healthcare providers in implementing these new standards of care, ultimately improving patient outcome and reducing disparities in treatment access. The bibliometric analysis of HER2-low breast cancer provides practical insights for clinicians and researchers. For clinicians, it highlights the growing importance of identifying HER2-low status, guiding treatment decisions and patient stratification, HER-2 immunohistochemical testing should employ two distinct antibody reagents on breast cancer specimens. This dual-reagent approach reduces false-negative risks associated with single-antibody assays, preventing misclassification of HER2-low patients as HER2-zero, thereby securing their eligibility for ADC-targeted therapies. For researchers, it reveals emerging trends and knowledge gaps, directing future studies towards exploring the biological mechanisms and optimizing targeted therapies. Priority should be given to: (1) Elucidating microenvironmental drivers of spatiotemporal HER-2 expression dynamics. (2) Advancing ctDNA-based multigene panel testing to replace repetitive tissue biopsies for real-time HER2 status monitoring. (3) Deciphering resistance mechanisms to ADCs and developing next-generation antibody-drug conjugates. This analysis underscores the need for standardized diagnostic criteria and emphasizes the potential of HER2-low as a therapeutic target, fostering collaboration between clinical practice and research to improve patient outcomes. Looking forward, the leveraging of these findings in Low- and Middle-Income Countries (LMICs) will be crucial for improving the management of HER2-low breast cancer. A major challenge lies in overcoming barriers to accessing advanced diagnostics and novel antibody-drug conjugates (ADCs). Nonetheless, our bibliometric findings—highlighting the focus on standardized diagnosis and treatment paradigms—provide a strategic roadmap for LMICs. Prioritizing the development and quality assurance of affordable diagnostic infrastructure (e.g., standardized immunohistochemistry) is the critical first step. Furthermore, our collaboration network analysis has identified key research institutions and leading experts in this field. Researchers from LMICs can utilize this “collaboration map” to proactively establish international partnerships with these core team, which would facilitate access to specialized technical training, promote sharing of critical research resources, and support the development of clinical studies.

This bibliometrics faces several limitations:1)Database Selection Bias: We select Web of Science Core Collection as the sole data source for this bibliometric analysis, while justified for its authoritative coverage of high-impact journals and citation network integrity, inherently introduces selection bias.2)citation-age effects༚While our bibliometric analysis captured key thematic shifts following the DESTINY-Breast04 (DB-04) trial, it may underestimate emerging trends due to citation lag—a phenomenon where novel significant findings require time to permeate the academic discourse and accumulate measurable impact.3)Restricting analysis to English-language articles excluded clinically valuable research in other languages. We selected 2018 as the study’s starting point because the ASCO/CAP guideline update issued that year first established a standardized diagnostic definition for HER2-low breast cancer (IHC 1 + or IHC 2+/ISH–). Prior to 2018, the absence of consensus diagnostic criteria precluded reliable identification of relevant literature in databases. Consequently, our retrieval strategy may have missed studies published before 2018 that retrospectively conform to the current HER2-low definition. A manual review of 56 pre-2018 papers reveals that only 2 papers [45, 46] satisfy the current HER2-low definition, justifying our choice of 2018 as starting point is meaningful. These issues highlight the need for more comprehensive and adaptable bibliometric approaches.

This bibliometric study utilized the “bibliometrix” package, VOSviewer, and CiteSpace to visually identify the countries, institutions, and authors that were making significant contributions to the field of HER2-low expression breast cancer. The study aimed to clarify the research hotspots and future trends in this area. However, it is important to note that the study had some limitations. These included the fact that it only includes articles from WoSCC, was limited to English language publications, and only considered articles and review articles. These limitations may introduce bias in the literature sources analyzed, and recent high-quality articles with low citation rates may not have a significant impact on the analysis results.

## Conclusion

This bibliometric analysis reveals a fast-growing body of research on HER2-low expression breast cancer. Diverging from HER2 zero or positive breast cancer, this particular subtype displays distinct molecular features, clinical results, and treatment strategies. By examining the distribution of literature worldwide on HER2-low expression breast cancer, this bibliometric study identifies cutting-edge research focuses and upcoming trends in the field, providing helpful insights for future investigators.

## Supplementary Information

Supplementary Material 1

Supplementary Material 2

Supplementary Material 3

Supplementary Material 4

Supplementary Material 5

Supplementary Material 6

Supplementary Material 7

Supplementary Material 8

Supplementary Material 9

## Data Availability

All data created or analyzed during this study are enrolled in this published article or are provided within the supplementary information files.

## References

[1] Smolarz B, Nowak AZ, Romanowicz H. Breast cancer-epidemiology, classification, pathogenesis and treatment (review of literature). Cancers (Basel). 2022. 10.3390/cancers14102569. 35626173 10.3390/cancers14102569 PMC9139759

[2] Tarantino P, Hamilton E, Tolaney SM, Cortes J, Morganti S, Ferraro E, et al. HER2-low breast cancer: pathological and clinical landscape. J Clin Oncol. 2020;38(17):1951–62. 32330069 10.1200/JCO.19.02488

[3] Fehrenbacher L, Cecchini RS, Geyer CE Jr., Rastogi P, Costantino JP, Atkins JN, et al. NSABP B-47/NRG oncology phase III randomized trial comparing adjuvant chemotherapy with or without trastuzumab in high-risk invasive breast cancer negative for HER2 by FISH and with IHC 1 + or 2. J Clin Oncol. 2020;38(5):444–53. 31821109 10.1200/JCO.19.01455 PMC7007289

[4] Trastuzumab. Deruxtecan in previously treated HER2-Low advanced breast cancer. N Engl J Med. 2022;387(1):9–20. 35665782 10.1056/NEJMoa2203690 PMC10561652

[5] Wolff AC, Hammond MEH, Allison KH, Harvey BE, Mangu PB, Bartlett JMS, et al. Human epidermal growth factor receptor 2 testing in breast cancer: American society of clinical oncology/college of American pathologists clinical practice guideline focused update. J Clin Oncol. 2018;36(20):2105–22. 29846122 10.1200/JCO.2018.77.8738

[6] Baselga J, Perez EA, Pienkowski T, Bell R. Adjuvant trastuzumab: a milestone in the treatment of HER-2-positive early breast cancer. Oncologist. 2006;11(Suppl 1):4–12. 16971734 10.1634/theoncologist.11-90001-4

[7] Ganz PA, Cecchini RS, Fehrenbacher L, Geyer CE Jr., Rastogi P, Crown JP, et al. NRG Oncology/NSABP B-47 menstrual history study: impact of adjuvant chemotherapy with and without trastuzumab. NPJ Breast Cancer. 2021;7(1):55. 34016989 10.1038/s41523-021-00264-2 PMC8137688

[8] Kang S, Kim SB. HER2-low breast cancer: now and in the future. Cancer Res Treat. 2024;56(3):700–20. 38291745 10.4143/crt.2023.1138 PMC11261208

[9] Mosele F, Deluche E, Lusque A, Le Bescond L, Filleron T, Pradat Y, et al. Trastuzumab deruxtecan in metastatic breast cancer with variable HER2 expression: the phase 2 DAISY trial. Nat Med. 2023;29(8):2110–20. 37488289 10.1038/s41591-023-02478-2 PMC10427426

[10] Hu XE, Yang P, Chen S, Wei G, Yuan L, Yang Z, et al. Clinical and biological heterogeneities in triple-negative breast cancer reveals a non-negligible role of HER2-low. Breast Cancer Res. 2023;25(1):34. 36998014 10.1186/s13058-023-01639-y PMC10061837

[11] Dai LJ, Ma D, Xu YZ, Li M, Li YW, Xiao Y, et al. Molecular features and clinical implications of the heterogeneity in Chinese patients with HER2-low breast cancer. Nat Commun. 2023;14(1):5112. 37607916 10.1038/s41467-023-40715-x PMC10444861

[12] Li M, Zheng A, Song M, Jin F, Pang M, Zhang Y, et al. From text to insight: a natural language processing-based analysis of burst and research trends in HER2-low breast cancer patients. Ageing Res Rev. 2025;106:102692. 39993452 10.1016/j.arr.2025.102692

[13] Deng S, Meng F, Wang L, Yang Z, Xuan L, Xuan Z, et al. Global research trends in non-muscle invasive bladder cancer: bibliometric and visualized analysis. Front Oncol. 2022;12:1044830. 36465379 10.3389/fonc.2022.1044830 PMC9713934

[14] Wang C, Zhang Y, Zhang Y, Li B. A bibliometric analysis of gastric cancer liver metastases: advances in mechanisms of occurrence and treatment options. Int J Surg. 2024;110(4):2288–99. 38215249 10.1097/JS9.0000000000001068 PMC11020032

[15] Meng X, Lu Z, Mi F, Sha S, Li T. Research hotspots and emerging trends in targeted therapy for colorectal cancer: a bibliometric analysis (2000–2023). Discover Oncol. 2025;16(1):789. 10.1007/s12672-025-02632-x PMC12084209 40380023

[16] van Eck NJ, Waltman L. Software survey: VOSviewer, a computer program for bibliometric mapping. Scientometrics. 2010;84(2):523–38. 20585380 10.1007/s11192-009-0146-3 PMC2883932

[17] Synnestvedt MB, Chen C, Holmes JH. CiteSpace II: visualization and knowledge discovery in bibliographic databases. AMIA Annu Symp Proc. 2005;2005:724–8. 16779135 PMC1560567

[18] Zhang H, Gao Y, Ying J, Yu H, Guo R, Xiong J, et al. Bibliometric analysis of global research on breast reconstruction after mastectomy for breast cancer from 2011 to 2021. J Cosmet Dermatol. 2023;22(7):2071–82. 36847708 10.1111/jocd.15683

[19] Qu F, Wang G, Wen P, Liu X, Zeng X. Knowledge mapping of immunotherapy for breast cancer: a bibliometric analysis from 2013 to 2022. Hum Vaccin Immunother. 2024;20(1):2335728. 38563136 10.1080/21645515.2024.2335728 PMC10989689

[20] Chen C. Science mapping: a systematic review of the literature. J Data Inf Sci. 2017;2(2):1–40.

[21] Aria M, Cuccurullo C, bibliometrix. An R-tool for comprehensive science mapping analysis. J Informetr. 2017;11(4):959–75.

[22] Ivanova M, Porta FM, D’Ercole M, Pescia C, Sajjadi E, Cursano G, et al. Standardized pathology report for HER2 testing in compliance with 2023 ASCO/CAP updates and 2023 ESMO consensus statements on HER2-low breast cancer. Virchows Arch. 2024;484(1):3–14. 37770765 10.1007/s00428-023-03656-w PMC10791807

[23] Tarantino P, Tolaney SM, Curigliano G. Trastuzumab deruxtecan (T-DXd) in HER2-low metastatic breast cancer treatment. Ann Oncol. 2023;34(10):949–50. 37499870 10.1016/j.annonc.2023.07.003

[24] Tarantino P, Gupta H, Hughes ME, Files J, Strauss S, Kirkner G, et al. Comprehensive genomic characterization of HER2-low and HER2-0 breast cancer. Nat Commun. 2023;14(1):7496. 37980405 10.1038/s41467-023-43324-w PMC10657399

[25] Tarantino P, Jin Q, Tayob N, Jeselsohn RM, Schnitt SJ, Vincuilla J, et al. Prognostic and biologic significance of ERBB2-Low expression in Early-Stage breast cancer. JAMA Oncol. 2022;8(8):1177–83. 35737367 10.1001/jamaoncol.2022.2286 PMC9227690

[26] Xia LY, Cao XC, Yu Y. Survival outcomes in HER2-low versus HER2-zero breast cancer after neoadjuvant chemotherapy: a meta-analysis. World J Surg Oncol. 2024;22(1):106. 38643188 10.1186/s12957-024-03382-w PMC11031865

[27] Nishimura R, Fujiki Y, Taira T, Miyaki T, Kanemitsu S, Yotsumoto D, et al. The clinicopathological and prognostic significance of HER2-Low breast cancer: a comparative analysis between HER2-Low and HER2-Zero subtypes. Clin Breast Cancer. 2024;24(5):431–8. 38472058 10.1016/j.clbc.2024.02.013

[28] Li H, Plichta JK, Li K, Jin Y, Thomas SM, Ma F, et al. Impact of HER2-low status for patients with early-stage breast cancer and non-pCR after neoadjuvant chemotherapy: a National Cancer Database analysis. Breast Cancer Res Treat. 2024;204(1):89–105. 38066250 10.1007/s10549-023-07171-z

[29] Zhu S, Lu Y, Fei X, Shen K, Chen X. Pathological complete response, category change, and prognostic significance of HER2-low breast cancer receiving neoadjuvant treatment: a multicenter analysis of 2489 cases. Br J Cancer. 2023;129(8):1274–83. 37604930 10.1038/s41416-023-02403-x PMC10575949

[30] Nicolò E, Boscolo Bielo L, Curigliano G, Tarantino P. The HER2-low revolution in breast oncology: steps forward and emerging challenges. Ther Adv Med Oncol. 2023;15:17588359231152842. 36844387 10.1177/17588359231152842 PMC9943960

[31] Amith SR, Fliegel L. Na(+)/H(+) exchanger-mediated hydrogen ion extrusion as a carcinogenic signal in triple-negative breast cancer etiopathogenesis and prospects for its inhibition in therapeutics. Semin Cancer Biol. 2017;43:35–41. 28104391 10.1016/j.semcancer.2017.01.004

[32] Schettini F, Chic N, Brasó-Maristany F, Paré L, Pascual T, Conte B, et al. Clinical, pathological, and PAM50 gene expression features of HER2-low breast cancer. NPJ Breast Cancer. 2021;7(1):1. 33397968 10.1038/s41523-020-00208-2 PMC7782714

[33] Banerji U, van Herpen CML, Saura C, Thistlethwaite F, Lord S, Moreno V, et al. Trastuzumab duocarmazine in locally advanced and metastatic solid tumours and HER2-expressing breast cancer: a phase 1 dose-escalation and dose-expansion study. Lancet Oncol. 2019;20(8):1124–35. 31257177 10.1016/S1470-2045(19)30328-6

[34] Marchiò C, Criscitiello C, Scatena C, Santinelli A, Graziano P, Malapelle U, et al. Think HER2 different: integrative diagnostic approaches for HER2-low breast cancer. Pathologica. 2023;115(6):292–301. 38180137 10.32074/1591-951X-942 PMC10767801

[35] Ergun Y, Ucar G, Akagunduz B. Comparison of HER2-zero and HER2-low in terms of clinicopathological factors and survival in early-stage breast cancer: a systematic review and meta-analysis. Cancer Treat Rev. 2023;115:102538. 36898351 10.1016/j.ctrv.2023.102538

[36] Yang C, Zhang X, Chen Y, Li P, Zhang J, Xu A, et al. Survival differences between HER2-0 and HER2-low-expressing breast cancer - a meta-analysis of early breast cancer patients. Crit Rev Oncol Hematol. 2023;185:103962. 36921780 10.1016/j.critrevonc.2023.103962

[37] Kang S, Lee SH, Lee HJ, Jeong H, Jeong JH, Kim JE, et al. Pathological complete response, long-term outcomes, and recurrence patterns in HER2-low versus HER2-zero breast cancer after neoadjuvant chemotherapy. Eur J Cancer. 2022;176:30–40. 36183652 10.1016/j.ejca.2022.08.031

[38] Viale G, Basik M, Niikura N, Tokunaga E, Brucker S, Penault-Llorca F, et al. Retrospective study to estimate the prevalence and describe the clinicopathological characteristics, treatments received, and outcomes of HER2-low breast cancer. ESMO Open. 2023;8(4):101615. 37562195 10.1016/j.esmoop.2023.101615 PMC10515285

[39] Hui T, Li S, Wang H, Ma X, Du F, Gao W, et al. An analysis of clinical and pathologic Features, recurindex genomic Profiles, and survival outcomes in HER2-Low breast cancer. Oncologist. 2023;28(12):e1160–9. 37279952 10.1093/oncolo/oyad159 PMC10712905

[40] Qu F, Lu R, Liu Q, Wu X, Huang X, Yin Y, et al. Antibody-drug conjugates transform the outcome of individuals with low-HER2-expression advanced breast cancer. Cancer. 2024;130(S8):1392–402. 38271367 10.1002/cncr.35205

[41] Ruan DY, Wu HX, Meng Q, Xu RH. Development of antibody-drug conjugates in cancer: overview and prospects. Cancer Commun (Lond). 2024;44(1):3–22. 38159059 10.1002/cac2.12517 PMC10794012

[42] Modi S, Park H, Murthy RK, Iwata H, Tamura K, Tsurutani J, et al. Antitumor activity and safety of trastuzumab Deruxtecan in patients with HER2-low-expressing advanced breast cancer: results from a phase Ib study. J Clin Oncol. 2020;38(17):1887–96. 32058843 10.1200/JCO.19.02318 PMC7280051

[43] Zhan M, Huang Z, Xu T, Xu X, Zheng H, Wu F. Cost-effectiveness analysis of trastuzumab deruxtecan in patients with HER2-low advanced breast cancer based on DESTINY-Breast04. Front Public Health. 2023;11:1049947. 37457280 10.3389/fpubh.2023.1049947 PMC10347396

[44] Subhan MA, Torchilin VP. Advances in targeted therapy of breast cancer with antibody-drug conjugate. Pharmaceutics. 2023. 10.3390/pharmaceutics15041242. 37111727 10.3390/pharmaceutics15041242 PMC10144345

[45] Collins D, Jacob W, Cejalvo JM, Ceppi M, James I, Hasmann M, et al. Direct estrogen receptor (ER) / HER family crosstalk mediating sensitivity to lumretuzumab and pertuzumab in ER + breast cancer. PLoS ONE. 2017. 10.1371/journal.pone.0177331. 28493933 10.1371/journal.pone.0177331 PMC5426757

[46] Datta J, Xu S, Rosemblit C, Smith JB, Cintolo JA, Powell DJ Jr, et al. CD4(+) T-helper type 1 cytokines and trastuzumab facilitate CD8(+) T-cell targeting of HER2/neu-expressing cancers. Cancer Immunol Res. 2015;3(5):455–63. 25791067 10.1158/2326-6066.CIR-14-0208 PMC4556111

